# Screening and analysis of proteins interacting with OsMADS16 in rice (*Oryza sativa* L.)

**DOI:** 10.1371/journal.pone.0221473

**Published:** 2019-08-22

**Authors:** Lan Kong, Yuanlin Duan, Yanfang Ye, Zhengzheng Cai, Feng Wang, Xiaojie Qu, Ronghua Qiu, Chunyan Wu, Weiren Wu

**Affiliations:** 1 Fujian Key Laboratory of Crop Breeding by Design, Fujian Agriculture and Forestry University, Fuzhou, Fujian, China; 2 Key Laboratory of Genetics, Breeding and Multiple Utilization of Crops, Ministry of Education, Fujian Agriculture and Forestry University, Fuzhou, Fujian, China; 3 Biotechnology Research Institute of Biotechnology, Fujian Academy of Agricultural Sciences, Fuzhou, Fujian, China; Chinese University of Hong Kong, HONG KONG

## Abstract

*OsMADS16*, a class B floral organ identity gene, plays a pivotal role in stamen formation in rice. To date, little is known about the interacting partners of OsMADS16 except for several MADS-box proteins. In this study, we constructed a high-quality cDNA library of young panicles (< 5 cm in length) and performed yeast two-hybrid (Y2H) screening using OsMADS16 as bait. Eleven candidate proteins interacting with OsMADS16 were identified by Y2H and validated by BiFC and Co-IP assays. Subcellular localization results further confirmed the possibility of the interactions of OsMADS16 with 10 of the candidate proteins in natural rice cells. Bioinformatics analysis indicated that these partners exerted various molecular, cellular and physiological functions. Some of them were known or likely to be related to reproductive events, such as stamen primordium initiation, differentiation and development (OsMADS2, OsMADS4 and OsCOP9) and pollen development (OsbHLH40 and Os6PGDH). Our results provide an important reference for further research on OsMADS16-mediated regulation mechanism on floral organ development and pollen formation.

## Introduction

Based on several studies on the floral organ development of eudicots, including *Arabidopsis thaliana*, *Petunia hybrida*, and *Antirrhinum majus*, the classical “ABC” model (later modified as the “ABCDE” model) was proposed to elucidate the genetic and molecular mechanism of floral meristem determinacy and floral organ identity [1–3]. In this model, the class A/B/C genes function individually or in combination with each other, leading to the formation of sepals, petals, stamens and carpels. Class A genes and class C genes define the development of sepals (whorl 1) and carpel (whorl 4), respectively; the combination of class A and class B genes determine the formation of petals (whorl 2); Class B genes act together with class C genes in specifying stamen identity (whorl 3) [4,5]. Furthermore, it has been demonstrated that almost all of these genes encode a family of eukaryotic transcription factors with a conserved MADS-box domain, which function through formation of homo-/hetero- multimeric complex [6,7].

The “ABC” model is also suitable for rice, although there are differences in the structure and developmental stages of flower between monocot and eudicot [8,9]. According to this model, several MADS-box genes have been identified and classified in rice [9–13]. For example, *OsMADS22*, classified into class A, specifies spikelet meristem determinacy in rice [14]. *OsMADS16*, belonging to class B, determines the specification of lodicules and stamens [12]. The class C gene *OsMADS3* plays a key role in stamen and ovule identity specification [15]. Additionally, class D and class E genes have been identified, such as *OsMADS13*, *OsMADS29*, *OsMADS1* and *OsMADS6* [16–19].

*OsMADS16* plays a central role in the development of whorl 2 and whorl 3 of rice flower. Ectopic expression of *OsMADS16* transforms pistil into a stamen-like organ [20], while mutation of *OsMADS16* (*spw1*) leads to the alternation of stamens to carpels and lodicules to palea-like organs [21]. Yeast two-hybrid (Y2H) assay and RNAi analysis showed that OsMADS16 interacts with either OsMADS2 or OsMADS4 to form the functional complexes, and OsMADS16-OsMADS4 heterodimer is required for the formation of ternary complexes [12,22]. A recent study showed that *OsMADS16* genetically interacts with *OsMADS3* and *OsMADS58* (two class C genes) in specifying floral patterning in rice. Both *spw1/osmads3* and *spw1/osmads58* double mutants display extra abnormal glume-like structure compared with *spw1* [23]. These findings revealed that OsMADS16 functions in specifying floral organ patterning via interacting with other floral homeotic genes.

However, there is rare information for other interacting partners of OsMADS16 except MADS-box proteins. Whether OsMADS16-mediated interactions are able to regulate other pathways during flower development remains unknown. In the present study, we identified eleven candidate partner proteins of OsMADS16 through Y2H, bimolecular fluorescence complementation (BiFC) and co-immunoprecipitation (Co-IP) assay, and demonstrated by bioinformatics analysis and subcellular localization assay that the interactions of OsMADS16 with its partners might exert distinct biological function mainly in nucleus. Our results will facilitate further research on OsMADS16-mediated regulation pathways of flower development.

## Materials and methods

### Materials

An *indica* rice variety Minghui-86 (MH86) was used for this study. Young panicles (< 5 cm in length) of MH86 were collected at booting stage and stored at -80°C.

### Construction of Y2H library

Total RNA was isolated from young panicles of MH86 using Trizol Reagent (Vazyme), and cDNA for library was gained from the total RNA using SMART^TM^ cDNA synthesis technology and amplified by long distance PCR (LD-PCR; Clontech, Cat. No.639201). Double-stranded cDNA was purified with CHROMA SPIN+TE- 400 Columns to eliminate <200 bp fragments (Clontech, Cat. No.630490). 2–5 μg of purified ds cDNA and 3 μg of pGADT7-Rec cloning vector were used for cotransformation of yeast competent cell Y187 according to the Yeastmaker Yeast Transformation System 2 protocol (Clontech, Cat. No.630439). Culture was plated on SD/-Leu agar plate. Library quality was calculated after incubation at 30°C for 3–5 d. Yeast cells were harvested and all colonies were pooled in YPDA liquid medium for Y2H library screening [24,25].

### Construction of OsMADS16 bait vector

To construct bait vector for the Y2H analysis, the full-length CDS of OsMADS16 was amplified with primers containing the restriction sites of *EcoR* I and *BamH* I (F: CGGAATTCATGGGGAGGGGCAAGATC, R: CGCGGATCCTCAACCGAGGCG CAGGT), and then cloned into bait vector pGBKT7 harboring GAL4 DNA-binding domain (BD). The correct recombination vector was introduced into yeast competent cell AH109 using PEG/LiAc-mediated method following the Yeastmaker Yeast Transformation System 2 protocol (Clontech, Cat. No.630439), and culture was spread on SD/-Trp plate. To test the self-activation of OsMADS16, yeast competent cell AH109 was cotransformed with bait vector pGBKT7-OSMADS16 and empty vector pGADT7 and selected on SD/Leu/-Trp/-His plate. The vectors pGBKT7-p53 and pGADT7-T were used as positive control, while pGBKT7-Lam and pGADT7-T were used as negative control.

### Screening of OsMADS16-interacting proteins

For screening of the proteins interacting with OsMADS16, the bait strain of pGBKT7-OsMADS16 was combined with yeast library cell in 2× YPDA liquid medium using yeast mating method followed by incubation at 30°C for 21–24 h according to the Matchmaker^®^ Gold Yeast Two-Hybrid System User Manual (Clontech, Cat. No.630489). The mating culture was plated on SD/-Leu/-Trp/-His agar plates for 7–14 d, and all the colonies were patched out onto higher stringency SD/-Leu/-Trp/-His/Ade agar plates. To further verify the interaction between them, the AD-Prey plasmids were rescued from yeast strain and sequenced. After eliminating false reading proteins, the full-length CDS of candidate proteins were amplified and cloned into prey vector pGADT7. Finally, yeast competent cell AH109 was cotransformed with the prey vector of each candidate OsMADS16-interacting protein and pGBKT7-OsMADS16, and selected on SD/-Leu/-Trp/-His and higher stringency SD/-Leu/-Trp/-His/Ade plates. The primers used are listed in S1 Table.

### BiFC assay

BiFC assay was performed to validate the protein interactions identified by the Y2H test. The coding sequences of *OsMADS16* and the genes of the candidate proteins were amplified and ligated into pCAMBIA1300S-YN and pCAMBIA2300S-YC, respectively, using ClonExpress^®^ II One Step Cloning Kit (Vazyme #C112). At least 10 μg mixed recombination plasmids were introduced into 100 μl rice protoplasts using the 40% PEG-mediated method [26,27]. The plasmids pCAMBIA1300S-YN-OsMADS16 and pCAMBIA2300S-YC were used as negative control. Fluorescence signals were observed using a confocal laser scanning microscopy (Leica, TCS, SP8). The primers used are listed in S1 Table.

### Co-IP assay

To further validate the interaction between OsMADS16 and interacted partners *in vivo*, Co-IP assay was conducted. The ORF of *OsMADS16* was amplified and inserted into pCAMBIA2300-Flag to generate the expression vector pCAMBIA2300-OsMADS16-Flag. Rice protoplasts from ten-day-old etiolated seedlings was cotransformed with the pCAMBIA2300-OsMADS16-Flag plasmid and different pCXSN-nGFP-candidate proteins. After incubation for 16 h, total proteins were extracted from protoplasts by lysis buffer (50 mM Tris-MES pH = 0.8, 0.5 M sucrose, 1 mM MgCl_2_, 10 mM EDTA, 1 mM PMSF, 1× protease inhibitor cocktail, 0.5 mM DTT, 0.1% Trixon-100), and combinated with 20 μl GFP-Nanoab-Agarose for 2 h at 4°C. The GFP-Nanoab-Agarose were washed four times in 0.5 ml lysis buffer, and resuspended in SDS sample buffer. The immunoprecipitation products were detected by SDS-PAGE and western blot using anti-Flag or anti-GFP antibody.

### Subcellular localization analysis

The coding sequences of the genes encoding the candidate OsMADS16-interacting proteins were amplified and inserted into the pCXSN-nGFP fusion vector, respectively. 5 μg of each construct was introduced into rice protoplasts using the 40% PEG-mediated method [26, 27]. The empty vector of pCXSN-nGFP was used as negative control. Fluorescence signals were observed as above. The primers used are listed in S1 Table.

Subcellular localization analysis was also performed using the tobacco cell system for two candidate interacting proteins, Os6PGDH and OsEXPB4. The fusion plasmids Os6PGDH-GFP and OsEXPB4-GFP and control plasmid were transformed into *Agrobacterium tumefaciens* strain GV3101. Recombinant *Agrobacterium tumefaciens* was suspended by suspension buffer (10 mM MgCl_2_, 10 mM, MES, 200 μM acetosyringone) and then injected into tobacco leaf. After 16 h, the GFP fluorescence was detected with a confocal laser scanning microscope. The primers used are listed in S1 Table.

## Results

### Y2H library constructed

**Fig 1:**
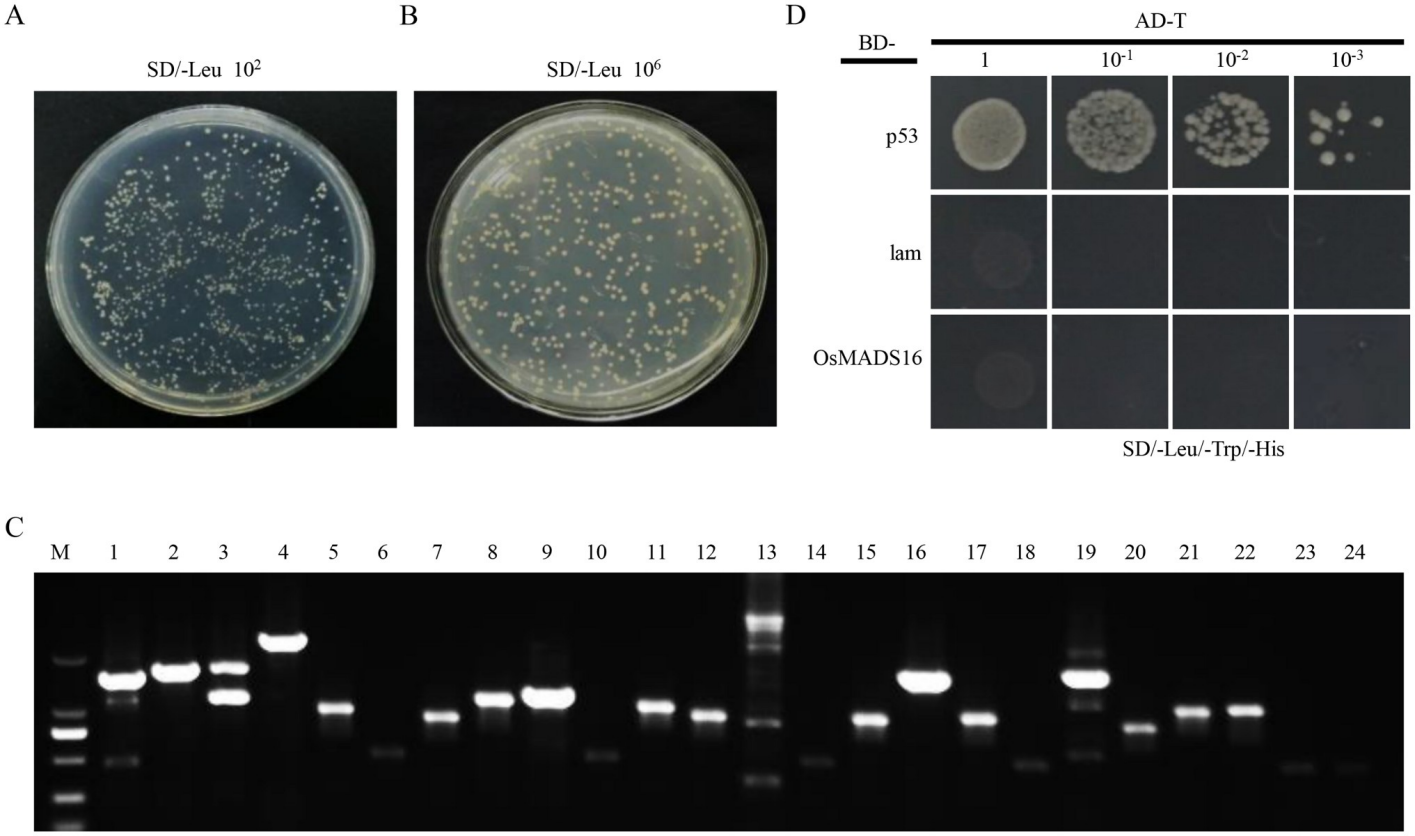
Examination of cDNA library quality and verification of OsMADS16 self-activation. A, estimation of the number of independent clones on a 10^2^ dilution SD/-Leu plate. B, estimation of the titer of cDNA library on a 10^6^ dilution SD/-Leu plate. C, PCR test of 24 random clones from the cDNA library. D, self-activation test of pGBKT7-OsMADS16.

A young panicle cDNA library of 50 ml in volume for Y2H analysis was constructed, which contained a total of ~5.4×10^6^ cfu independent clones (Fig 1A), with a titer of ~8.7×10^8^ cfu/ml (Fig 1B), exceeding the general requirement of library titer (2×10^7^ cfu/ml). The recombination efficiency was 98.5%. Examination of 24 randomly selected colonies indicated that the insert size of the library ranged from 0.3 to 2.0 kb (Fig 1C). These results indicated that the library was of high-quality and could be used for screening of OsMADS16-interaction proteins.

### Potential OsMADS16-interacting partners identified by Y2H

It is reported that the C-terminal of OsMADS16 has transcription activation ability [6, 21]. Therefore, in order to perform Y2H analysis, we first examined whether OsMADS16 could activate itself. The complete ORF of *OsMADS16* was inserted into the BD vector as bait and tested on a selective medium SD/-Leu/-Trp/-His. The result showed that the corresponding clones were unable to grow in the medium lacking histidine, indicating that OsMADS16 did not have the property of self-activation (Fig 1D).

**Fig 2:**
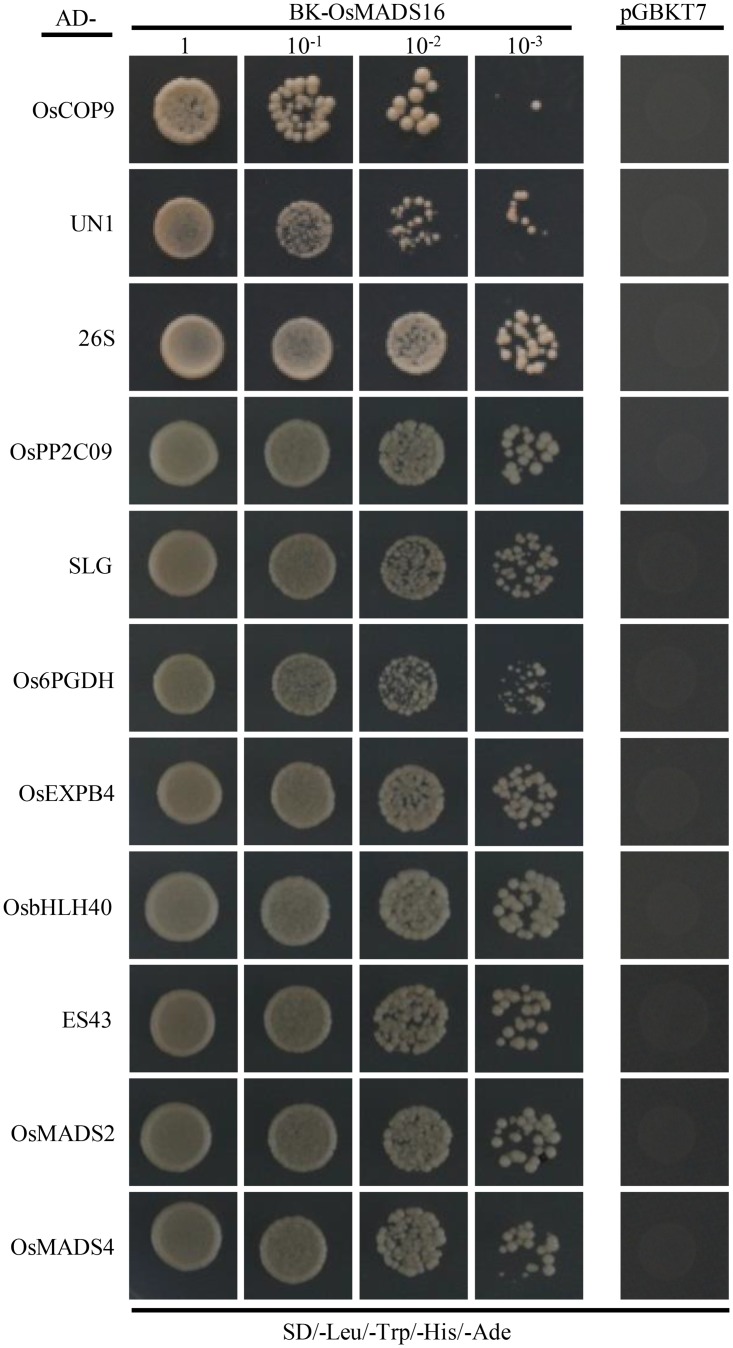
Examination of interactions between pGBKT7-OsMADS16 and pGADT7-candidate proteins on SD/-Leu/-Trp/-His/-Ade plates.

A total of 86 clones were initially screened from the cDNA library on a higher stringency medium lacking histidine and adenine. After sequencing analysis and auto-activation testing, only 24 genes were obtained, some of which were present with only truncated sequences. To further investigate the interactions of OsMADS16 with these proteins, we cloned their entire ORF sequences and re-examined by Y2H. Finally, 11 proteins displaying interaction with OsMADS16 were identified, including OsMADS2, OsMADS4, OsCOP9, OsbHLH40, Os6PGDH, OsPP2C09, and so on (Fig 2), which were taken as the candidate partners of OsMADS16.

### Verification of the interactions between OsMADS16 and candidate partners

**Fig 3:**
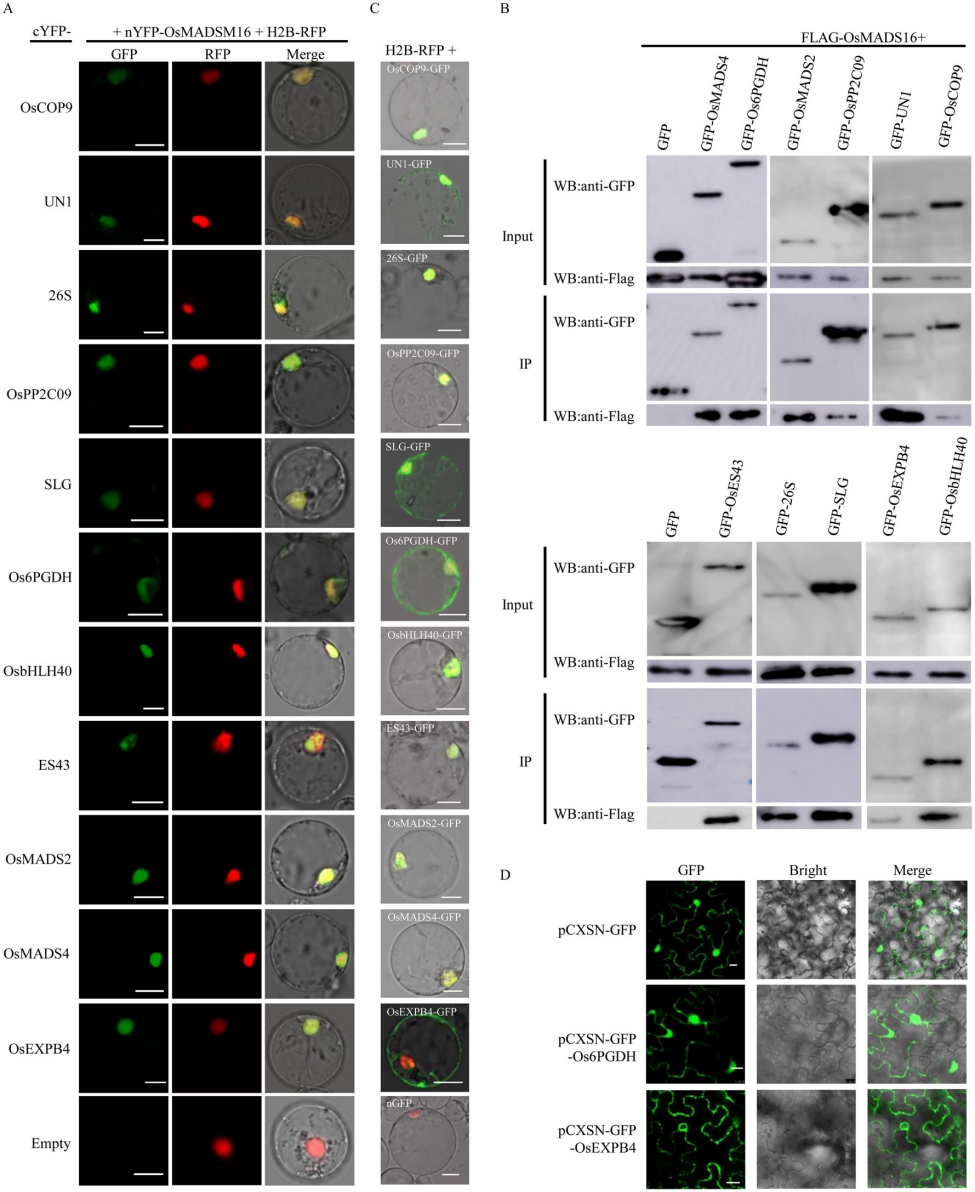
OsMADS16 interaction with 11 candidate partners. A and B, BiFC (A) and Co-IP (B) assays of the interactions between OsMADS16 and 11 candidate partners in rice protoplast. H2B was used as nuclear co-localization marker. C, Subcellular localization assay of 11 candidate proteins in rice protoplast. D, Subcellular localization assay of Os6PGDH and OsEXPB4 in tobacco cell. Bar = 10 μm.

BiFC and Co-IP assays were carried out in rice protoplasts to verify the interactions of OsMADS16 with the candidate partners identified by Y2H. In the BiFC assay, YFP fluorescence signals were observed to be accumulated at nucleus for all the 11 candidate partners, overlapping with the nuclear co-localization marker H2B, while no fluorescence was observed in the negative control presented with nYFP-OsMADS16 and cYFP-empty (Fig 3A). In the Co-IP assays, the protein complexes pulled down by GFP-Nanoab-Agarose were recognized by anti-Flag antibody in all the lines cotransformed with Flag-OsMADS16 and GFP-candidate proteins (Fig 3B). Both of the BiFC and the Co-IP results validated that OsMADS16 can physically interact with all the candidate partners. In addition, the BiFC results also suggested that the interactions all occur in nucleus.

To examine the possibility that these interactions can occur in natural rice cells, we performed subcellular localization assay on the candidate partners in rice protoplasts. The results showed that 10 of the candidate partners (excluding OsEXPB4) were localized specifically or dominantly in nucleus (Fig 3C). The localization of Os6PGDH in nucleus was also shown in tobacco cells (Fig 3D). These results were consistent with the BiFC results (Fig 3A) and overlapped with that of OsMADS16 as a transcription factor, suggesting that interactions between OsMADS16 and these 10 proteins are possible in real situations.

OsEXPB4 is predicted to be an expansin B4-like protein or a cell wall protein. The subcellular localization results in rice protoplasts indicated that OsEXPB4 was mainly located on cell membrane (Fig 3C) with only a few cases of OsEXPB4-GFP signal detected in nucleus (S1 Fig). The localization of OsEXPB4-GFP in nucleus was not observed in tobacco cells, where OsEXPB4-GFP was clearly shown to be located on cell membrane (including nuclear membrane) and possibly on cell wall as well (Fig 3D). Therefore, the possibility of interaction between OsMADS16 and OsEXPB4 in natural rice cells is doubtful. For this reason, we removed OsEXPB4 from the list of the candidate partners of OsMADS16.

### Annotated functions of candidate proteins

Bioinformatics analysis indicated that the rest 10 candidate proteins interacting with OsMADS16 exerted a series of molecular, cellular, and physiological functions (Table 1), including binding with DNA and regulating gene expression (OsMADS2, OsMADS4, OsbHLH40 and ES43), regulating cell cycle and protein modification (OsCOP9), involved in cell division (Os6PGDH), acting as an ABA signal regulator and responding to abiotic stress (OsPP2C09), servicing as a proteasome regulatory subunit involved in protein degradation (26S), and correlated with grain size (SLG).

**Table 1:** Information of OsMADS16-interacting genes identified.

No.	Gene locus	Gene symbols	Fragment size (bp)	Potential function	Location
1	Os01g0883100	*OsMADS2*	912	PISTILLATA-like MADS box protein, regulation of lodicule formation	nucleus
2	Os05g0423400	*OsMADS4*	893	PISTILLATA-like MADS box protein, regulation of lodicule and stamen formation	nucleus
3	Os03g0260600	*OsbHLH40*	851	Sequence-specific DNA binding transcription factor activity	nucleus
4	Os08g0421900	*ES43*	709	Chromatin remodeling protein EBS isoform X1; DNA binging; flower development	nucleus
5	Os08g0119000	*OsCOP9*	603	COP9 complex subunit 3; cell cycle; protein modification process, flower developmemt	nucleus
6	Os01g0112100	*UN1*	419	Uncharacterized protein	cytosol, nucleus
7	Os02g0199900	*26S*	892	ATP-dependent 26S proteasome regulatory subunit; nucleotide binding	nucleus
8	Os01g0846300	*OsPP2C09*	313	Protein serine/threonine phosphatase 2C, response to abiotic stimulus	nucleus
9	Os08g0562500	*SLG*	706	BAHD acyltransferase-like protein gene; Regulate grain size; embryo development	cytosol, nucleus
10	Os06g0111500	*Os6PGDH*	779	Phosphogluconate dehydrogenase activity; cell division, pollen development	cytosol, nucleus

## Discussion

Y2H and GFP-based techniques have been widely employed to study the functions and molecular mechanisms of proteins. In the present study, based on Y2H screening, BiFC and Co-IP validations, we identified 10 proteins interacting with OsMADS16 (Figs 2 and 3). Among them, two proteins (OsMADS2 and OsMADS4) have been found to interact with OsMADS16 before [12,22]. The heterodimer of OsMADS16 and OsMADS2/OsMADS4 is essential for floral organ identity determination [12]. Moreover, BiFC and subcellular localization analyses showed that most of the partner proteins and their interactions with OsMADS16 were mainly localized in nucleus (Fig 3A and 3B), suggesting that the OsMADS16-mediated pathways primary occur in nucleus. This is consistent with the feature of OsMADS16. Therefore, the results of this study appeared to be reasonable and acceptable.

Except OsMADS2 and OsMADS4, all the other partner proteins of OsMADS16 identified in this study are new and have quite diverse functions (Table 1), implying that OsMADS16 might also exert other biological functions associated with flower development. One of the proteins worth noting is OsCOP9 (Table 1). COP9 signalosome (CSN) is a multiprotein complex, which participates in several developmental pathways. Absence of COP9 leads to cell cycle arrest in *Saccharomyces pombe* [28]. In *Arabidopsis*, COP9 is highly expressed in floral tissues and regulates the development of petal and stamen by positively modulating SCF^UFO^-mediated AP3 activation [29,30]. OsMADS16 is the unique orthologue of AP3 in rice and displays the same function [21]. Therefore, the interaction between OsMADS16 and OsCOP9 implied that a similar mechanism might probably exist in rice.

Studies have shown that some MADS-box genes are involved in pollen fertility [31–33]. In *Arabidopsis*, *AG* (class C gene), along with *PI* and *AP3*, controls microsporogenesis via regulation of *SPOROCYTELESS/NOZZLE* (*SPL/NZZ*) [34]. In rice, *OsMADS62i/Osmads63* and *OsMADS68i/Osmads63* transgenic lines exhibit abnormal pollen phenotypes, suggesting that *OsMADS62*, *OsMADS63* and *OsMADS68*, which are specifically expressed in pollen, may be involved in pollen development [35]. In addition, transgenic tall fescue introduced with chimeric repressors of rice *SPW1* and *OsMADS58* produces indehiscent anther without pollens [36]. Interestingly, two OsMADS16-partner proteins identified in this study, OsbHLH40 and Os6PGDH (Table 1), were either associated with or potentially related to pollens. OsbHLH40 is a member of the basic helix-loop-helix (bHLH) transcription factor family. Four rice bHLH proteins have been found to be associated with tapetum development and programmed cell death (PCD), including UDT1 (bHLH164), TDR1 (bHLH5), EAT1/DTD1 (bHLH141) and bHLH142 [37–39]. Recently, Huang et al. showed that the expression of Os6PGDH (6-phosphogluconate dehydrogenase) is significantly higher in inflorescence than that in roots, leaves and embryos, suggesting that Os6PGDH is closely related to cell division [40]. Liang et al. pointed out that decreased activity of Os6PGDH affects the operation of pentose phosphate pathway and causes deficiency of ribose-5-phosphate, leading to pollen abortion of CMS lines [41]. The above information implies that OsMADS16 might probably also plays a role in pollen development.

ABA plays an important role in plant development, such as seed dormancy, germination and reproduction [42]. Recent research uncovered that the balance of ABA level in vivo is important for reproductive stage stress tolerance in cereals [43]. OsPP2C09 identified in this study (Table 1) could bind to ABA receptors (OsPYLs) and then be inhibited in ABA signaling pathway. In normal condition, PP2C proteins bind and inhibit the protein kinases (SnRK2), which activate the expression of ABA-responsive genes, but the inhibition is counteracted by OsPYLs-OsPP2Cs upon ABA treatment [43–45]. The interaction of OsMADS16 with OsPP2C09 implied that it might be also involved in ABA-related pathways.

It has been reported that Os6PGDH is located in cytoplasm, involved in an oxidative pentose phosphate pathway in cytosol [46]. The results of this study, however, clearly showed that Os6PGDH also existed in nucleus (Fig 3B and 3C). As there is no evidence that an additional oxidative pentose phosphate pathway may exist in the nucleus, the results implied that Os6PGDH might have a new unknown molecular function in nucleus. Since it was shown in this study that Os6PGDH can interact with OsMADS16, whether the unknown function of Os6PGDH in nucleus is associated with OsMADS16 is an interesting issue to be clarified.

In summary, the results of this study suggested that OsMADS16 could interact with a number of proteins with diverse functions, implying that it might exert diverse physiological functions associated with floral organ development and pollen formation. Further studies based on these proteins would provide new insights into the mechanisms of how OsMADS16 influences floral organ development and pollen formation in rice.

## Supporting information

S1 Table PCR primers used in this study. (DOCX)

S1 Fig Subcellular localization assay of OsEXPB4 in rice protoplasts. (PDF)
