# Supplementary material for: Screening and analysis of proteins interacting with OsMADS16 in rice (Oryza sativa L.)
Source: PLoS One. 2019 Aug 22;14(8):e0221473. doi: 10.1371/journal.pone.0221473 (PMC6705763; doi:10.1371/journal.pone.0221473)
Supplement: S1 Table — (DOCX) [file pone.0221473.s001.docx]

**S1 Table. PCR primers used in this study**

| **Primer pair name** | **Forward primer** | **Reverse primer** |
| --- | --- | --- |
| pGBKT7-OsMADS16 | CGGAATTCATGGGGAGGGGCAAGATC | CGCGGATCCTCAACCGAGGCGCAGGT |
| pGADT7-OsMADS4 | CGGAATTCATGGGGCGCGGCAAG | CGCGGATCCCTACTTCTCCTGCTGGAG |
| pGADT7-OsMADS2 | GGAATTCCATATGATGGGGCGCGGGAA | CGGAATTCTTAATTGTTCTCCTG |
| pGADT7-OsbHLH40 | CGGAATTCATGGGTGCTCATG | CGCGGATCCTTAGATAGACCTG |
| pGADT7-ES43 | CGGAATTCATGGCCAAGACCA | CGCGGATCCTCACCTCCTCTGCCTT |
| pGADT7-OsCOP9 | CGGAATTCATGGAGACGGTGGAGACG | CGCGGATCCTCAAAAATATTTGTGCGG |
| pGADT7-UN1 | CGGAATTCATGACGCTCCTCGCCGCC | CGCGGATCCTCACACATCTTGCTGCAT |
| pGADT7-26S | CGGAATTCATGGCCGACGGGGAGGA | CGCGGATCCTTACTCTTTGCCGAAGTC |
| pGADT7-OsPP2C09 | CGGAATTCATGGCCGAGATCTGCTGC | CGCGGATCCTCACAATCCCCGGCGGAC |
| pGADT7-SLG | CGGAATTCATGGCGGCCGTCGACAAT | CGCGGATCCTTAAGGGGCTCGCATTTG |
| pGADT7-Os6PGDH | CGGAATTCATGGCTGTCACTAGAATT | CGCGGATCCTCACATCTTAGCAGCACG |
| pGADT7-OsEXPB4 | CGGAATTCATGGGCTCGCTGTCCTGT | CGCGGATCCTCAGCTGTACTGGACGAAG |
| YN-OsMADS16 | GATTTCTGAGGAGGATCTTCCCGGGATGGGGAGGGGCAAGATCGA | GCAGGGCATGCCTGCAGGTCGACTCAACCGAGGCGCAGGTCGT |
| YC-OsMADS4 | CGACTCTAGGAGCTCGGTACCCGGGATGGGGCGCGGCAAG | GGAACATCGTATGGGTACATACTAGTCTTCTCCTGCTGGAG |
| YC-OsMADS2 | CGACTCTAGGAGCTCGGTACCCGGGATGGGGCGCGGGAAG | GGAACATCGTATGGGTACATACTAGTATTGTTCTCCTGCAG |
| YC-OsbHLH40 | CGACTCTAGGAGCTCGGTACCCGGGATGGGTGCTCATGGAGACCA | GGAACATCGTATGGGTACATACTAGTGATAGACCTGTTCTCTAGGAT |
| YC-ES43 | CGACTCTAGGAGCTCGGTACCCGGGATGGCCAAGACCAAGCAGGG | GGAACATCGTATGGGTACATACTAGTCCTCCTCTGCCTTTTTGACT |
| YC-OsCOP9 | CGACTCTAGGAGCTCGGTACCCGGGATGACGCTCCTCGCCGCC | GGAACATCGTATGGGTACATACTAGTCACATCTTGCTGCAT |
| YC-UN1 | CGACTCTAGGAGCTCGGTACCCGGGATGACGCTCCTCGCCGCC | GGAACATCGTATGGGTACATACTAGTCACATCTTGCTGCAT |
| YC-26S | CGACTCTAGGAGCTCGGTACCCGGGATGGCCGACGGGGAGGAC | GGAACATCGTATGGGTACATACTAGTCTCTTTGCCGAAGTC |
| YC-OsPP2C09 | CGACTCTAGGAGCTCGGTACCCGGGATGGCCGAGATCTGCTGC | GGAACATCGTATGGGTACATACTAGTCAATCCCCGGCGGAC |
| YC-SLG | CGACTCTAGGAGCTCGGTACCCGGGATGGCGGCCGTCGACAAT | GGAACATCGTATGGGTACATACTAGTAGGGGCTCGCATTTG |
| YC-Os6PGDH | CGACTCTAGGAGCTCGGTACCCGGGATGGCTGTCACTAGAATT | GGAACATCGTATGGGTACATACTAGTCATCTTAGCAGCACG |
| YC-OsEXPB4 | CGACTCTAGGAGCTCGGTACCCGGGATGGGCTCGCTGTCCTGT | GGAACATCGTATGGGTACATACTAGTGCTGTACTGGACGAAG |
| OsMADS16Sub | TATGGGGAGGGGCAAGATCGA | TCAACCGAGGCGCAGGTCGT |
| OsMADS4Sub | CTGGGGCGCGGCAAG | CTACTTCTCCTGCTGGAG |
| OsMADS2Sub | CATGGGGCGCGGGAA | TTAATTGTTCTCCTG |
| OsbHLH40Sub | CATGGGTGCTCATGGAGACCA | TTAGATAGACCTGTTCTCTA |
| ES43Sub | CATGGCCAAGACCAAGCAGGG | TCACCTCCTCTGCCTTTTTG |
| OsCOP9Sub | CATGGAGACGGTGGAGACG | TCAAAAATATTTGTGCGG |
| UN1Sub | CATGACGCTCCTCGCCGCC | TCACACATCTTGCTGCAT |
| 26SSub | CATGGCCGACGGGGAGGA | TTACTCTTTGCCGAAGTC |
| OsPP2C09Sub | CATGGCCGAGATCTGCTGC | TCACAATCCCCGGCGGAC |
| SLGSub | CATGGCGGCCGTCGACAAT | TTAAGGGGCTCGCATTTG |
| Os6PGDHSub | CATGGCTGTCACTAGAATT | TCACATCTTAGCAGCACG |
| OsEXPB4Sub | CATGGGCTCGCTGTCCTGT | TCAGCTGTACTGGACGAAG |
| M16-Flag | CGCGGATCCATGGGGAGGGGCAAGA | AAAAGGCCTTTTACCGAGGCGCAGGT |

Note: The underlined sequences are cleavage sites of restriction enzymes and homologous recombination linkers
