# Supplementary figures and images for: Screening and analysis of proteins interacting with OsMADS16 in rice (Oryza sativa L.)
Source: PLoS One. 2019 Aug 22;14(8):e0221473. doi: 10.1371/journal.pone.0221473 (PMC6705763; doi:10.1371/journal.pone.0221473)

pCXS<sub>N</sub>-GFP-OsEXPB4

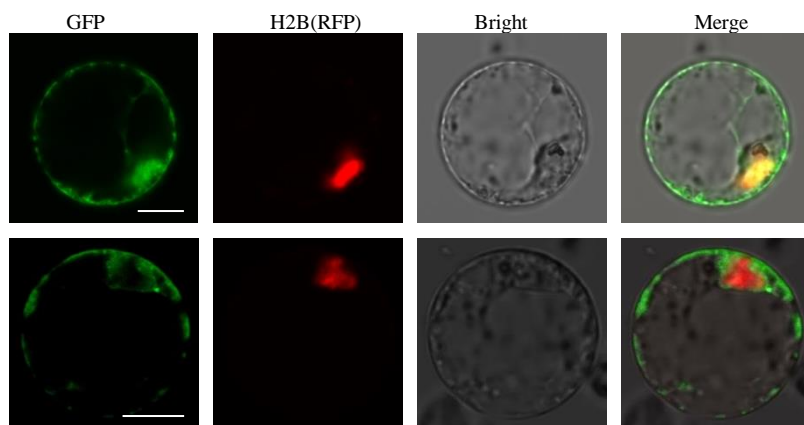

Supplement: S1 Fig — (PDF) [file pone.0221473.s002.pdf]
